# Serum procalcitonin in the diagnosis of pneumonia in the neurosurgical intensive care unit

**DOI:** 10.1007/s10143-025-03529-7

**Published:** 2025-04-21

**Authors:** Manou Overstijns, Pierre Scheffler, Jürgen Buttler, Jürgen Beck, Amir El Rahal

**Affiliations:** 1 Department of Neurosurgery, Medical Center University of Freiburg, Freiburg im Breisgau, 79106 Germany; 2 Faculty of Medicine, University of Geneva, Geneva, Switzerland

**Keywords:** Procalcitonin, Pneumonia, Intensive care units, Neurologic hospital-acquired infections

## Abstract

Procalcitonin (PCT) is a biomarker for bacterial infections, with controversial utility in diagnosing hospital-acquired pneumonia (HAP) in neurosurgical intensive care unit (NICU) patients. Establishing an optimal PCT cutoff value could enhance diagnostic accuracy. This retrospective single-center study included NICU patients hospitalized between January 1, 2021, and December 31, 2022, who underwent routine serum PCT measurement. HAP was diagnosed based on clinical, biochemical, microbiological, and radiological data. The optimal PCT cutoff value was identified using the Youden Index. Associations between PCT levels, radiological findings, sputum cultures, and confirmed HAP were analyzed using chi-square tests. A multivariate logistic regression was performed to identify independent predictors of elevated PCT. Among 2363 patients, 193 met inclusion criteria, and 148 were diagnosed with HAP. The optimal PCT cutoff value was 0.095 ng/mL, yielding a sensitivity of 89.2% and specificity of 93.3% (*p* < 0.001). This cutoff resulted in a positive likelihood ratio of 13.3 and a negative likelihood ratio of 0.116. Radiological signs of pneumonia and positive sputum cultures were observed in 48.4% and 78.4% of HAP cases, respectively, but neither showed a significant association with HAP (*p* = 0.135 and *p* = 0.056). Leukocytosis was significantly associated with HAP but had low specificity, while CRP showed a non-significant trend. In multivariate analysis, only confirmed HAP independently predicted PCT elevation. PCT, with a cutoff value of 0.095 ng/mL, shows high diagnostic accuracy for HAP in NICU patients and could enhance early identification and treatment. Our findings suggest that elevated PCT is primarily driven by HAP rather than non-infectious inflammatory triggers such as trauma or recent surgery. Further prospective studies are warranted to validate these findings.

## Clinical trial number

Not applicable

## Introduction

Procalcitonin (PCT) is a protein and calcitonin precursor [1]. PCT plasma concentration is quantitatively upregulated with an inflammatory reaction in response to TNF-alpha, endotoxins and IL-6^2^. In uninfected individuals, PCT is produced almost entirely within the thyroid C cells and converted to calcitonin before PCT enters systemic circulation. Therefore, healthy individuals typically demonstrate very low levels of serum PCT (< 0.02 ng/ml)^2^. In contrast, patients with bacterial infections produce PCT via an alternative pathway in nonthyroidal tissue types, including the spleen, kidney, adipocytes, pancreas, colon, brain, and lungs [3]. Such parenchymal tissue lacks the processing pathway necessary to convert PCT to calcitonin, resulting in PCT entering the blood circulation and subsequently raising the serum PCT level.

Patients in neurosurgical intensive care units (NICUs) are at an increased risk of developing hospital-acquired pneumonia (HAP) due to factors such as altered levels of consciousness, impaired protective airway reflexes, mechanical ventilation, and the invasive nature of neurosurgical procedures that can compromise the immune system and respiratory function [4]. The incidence of HAP in NICUs is notably higher compared to other ICUs, and early diagnosis is critical to initiate timely antibiotic therapy and improve patient outcomes [5]. Diagnosing HAP in neurosurgical patients is challenging because neurological deficits and intensive care treatments complicate the clinical examination process. Additionally, routine inflammatory markers such as C-reactive protein (CRP) are often pathologically increased postoperatively due to trauma or, in the case of an intracranial hemorrhage [6].

The diagnostic utility of PCT is still controversial in the literature. Although the utilization of PCT in guiding antimicrobial therapy for patients with HAP has been extensively described, other studies showed no clear benefit of PCT in diagnosing HAP [7–9]. Furthermore, in the neurosurgical intensive care unit (NICU) specifically, a limited utility of PCT with poor diagnostic value is described [9]. Currently, there needs to be more consensus on the optimal cutoff value of PCT for diagnosing HAP in NICU patients. Standardizing this cutoff is crucial because using inappropriate thresholds can lead to misdiagnosis, either missing cases of pneumonia or prompting unnecessary antibiotic use, contributing to antibiotic resistance [10]. This study aimed to determine the optimal serum PCT cut-off value and investigate the accuracy of this PCT cut-off value in the diagnosis of HAP in patients in the NICU.

## Methods

### Patient selection

We conducted a retrospective cohort study at the Department of Neurosurgery, Medical Center University of Freiburg, located in Freiburg in Breisgau, Germany, approved by the local ethics committee. The electronic health record was searched for patients in the NICU hospitalized between 01.01.2021 and 31.12.2022. Inclusion criteria were: (1) Patients 18 years or older and (2) Patients who underwent procalcitonin assays due to the suspicion of HAP. Exclusion criteria were: (1) First serum Procalcitonin was determined > 24 h after the start of HAP symptoms (2) Procalcitonin was determined for a reason other than suspected HAP (3) Patient was already under antibiotic therapy when PCT was determined.

### Procalcitonin assay

Serum PCT levels were measured using the Thermo Scientific™ B·R·A·H·M·S PCT™ sensitive KRYPTOR™, which has a functional sensitivity of 0.02 ng/mL and an inter-assay coefficient of variation of less than 5%. Blood samples were collected as part of routine laboratory assessments, typically conducted every 48 h, regardless of clinical suspicion of infection. During the study period, the optimal PCT cutoff value for diagnosing HAP was not established in our NICU, and PCT results were not routinely reviewed by clinicians at the time of making treatment decisions. During the study period, PCT values were not routinely reviewed by treating physicians at the time of clinical decision-making. The initiation of antibiotic therapy followed institutional standard protocols and was not based on PCT results. Empirical treatment was initiated with piperacillin–tazobactam, and antibiotics were subsequently adjusted based on available sputum antibiograms. Antibiotic decisions were made based on clinical judgment, incorporating symptoms, general laboratory markers (excluding PCT), and radiological findings when present.

### Definition of Hospital-acquired pneumonia

Hospital-acquired pneumonia (HAP) was defined in our study according to the Centers for Disease Control and Prevention (CDC) guidelines as pneumonia that develops 48 h or more after hospital admission and was not present or incubating at the time of admission [11]. To confirm a diagnosis of HAP, we required the following criteria:

- **Initiation of antibiotic therapy** by the treating physician based on clinical judgment.
- **At least two of the following clinical signs or symptoms suggestive of pneumonia.**

-  Fever exceeding 38 °C.
-  Purulent sputum or increased respiratory secretions.
-  New or worsening cough.
-  Dyspnea (shortness of breath) or tachypnea (rapid breathing).
-  Hypoxia indicated by a PaO₂/FiO₂ ratio less than 300 mmHg.
-  Auscultatory findings consistent with pneumonia such as crackles or bronchial breath sounds.

- **Laboratory findings supporting the diagnosis.**

-  Leukocytosis, defined as a white blood cell count greater than 12,000 cells/mm³.

Radiological and microbiological evidence were incorporated into the diagnosis of HAP when available. Radiological evidence was obtained from chest radiographs demonstrating new or progressive infiltrates. Radiological images were interpreted by a rotating team of board-certified radiologists as part of standard clinical care. However, recognizing that chest imaging has been proven to have low sensitivity in detecting pneumonia, especially in critically ill patients, our diagnosis did not rely solely on radiographic findings [12]. Instead, radiological results were considered in conjunction with clinical signs and laboratory findings to strengthen the diagnostic accuracy. Similarly, microbiological evidence was utilized when available to support the diagnosis. This included positive cultures from respiratory secretions such as sputum, endotracheal aspirate, or bronchoalveolar lavage.

### Data collection

Data was accessed on 01.07.2024, the authors had access to information that could identify individual participants during data collection. The local ethical committee waived the need for consent. Data collection included age, sex, admission diagnosis, positive sputum culture, radiological evidence of pneumonia, length of stay in the NICU, intubation status and mechanical ventilation, creatinine values, Glasgow Coma Scale (GCS) score at admission, and whether the patient had recently undergone surgery (< 10 days). Clinical and laboratory data relevant to the diagnosis of HAP were recorded, including presence of leukocytosis, defined as a leukocyte count above 9.8 × 10³/µL for males and 10.4 × 10³/µL for females, based on institutional laboratory standards. Confirmation of pneumonia diagnosis was based on clinical signs (fever, yellow phlegm production, increasing supplemental oxygen, crepitations during auscultation) and Laboratory results (leukocytosis, neutrophil left shift), which resulted in the initiation of antibiotic therapy. Although CRP values were available in a small number of patients (*n* = 31), they were not routinely collected in our NICU and thus not included in the multivariate analysis due to high false-positive rates in the neurosurgical patient-population [6].

### Statistical analysis

Statistical analyses were performed using SPSS Statistics version 25.0 (IBM Corp., Armonk, NY, USA). Data distribution was assessed using the Shapiro-Wilk test.

The diagnostic performance of serum PCT levels was evaluated using receiver operating characteristic (ROC) curve analysis, comparing patients with confirmed HAP to those without. The area under the ROC curve (AUC) was calculated to assess discrimination ability. The optimal PCT cutoff value was determined using the Youden Index, which maximizes the sum of sensitivity and specificity.

Associations between the optimal PCT cutoff value, radiological findings, sputum culture results, and confirmed HAP were analyzed using chi-square tests or Fisher’s exact test, as appropriate. To assess whether the age or length of stay differed between patients with and without HAP, we used the Mann-Whitney U test, as the data were not normally distributed. A p-value of less than 0.05 was considered statistically significant. Missing data were handled using pairwise deletion for statistical tests, assuming data were randomly missing.

In addition, an univariate and multivariate logistic regression analysis was performed to evaluate independent predictors of serum procalcitonin above the optimal PCT cutoff value. Variables included in the model were selected based on clinical relevance and literature review. As CRP values were only available in a limited subset of patients, they were not included in the multivariate model due to the small sample size and institutional practice of not routinely measuring CRP in this population.

## Results

### Demography

Of the 2363 patients in our NICU, 193 met the inclusion criteria, of which 148 patients were diagnosed with HAP (Table 1). The median age of all the patients was 71 years (IQR 60–81 years). The median length of stay was 19 days (IQR 11–29 days).

**Table 1:** Baseline characteristics of study participants

Characteristic	Total (*n* = 193)	HAP Group (*n* = 148)	Non-HAP Group (*n* = 45)	*p*-value		
**Age (years)**						
- Median (IQR)	71 (IQR: 60–81)	72 (IQR: 61–82)	68 (IQR: 56–74)	0.419		
**Gender**,** n (%)**				0.842		
- Male	110 (57.0%)	25 (55.6%)	85 (57.4%)			
- Female	83 (43.0%)	20 (44.4%)	63 (42.6%)			
**Length of NICU stay (days)**				0.227		
- Median (IQR)	19 (IQR: 11–29)	19 (IQR: 11–29.8)	16 (IQR: 9.75–26.25)			
**PCT level (ng/ml)**				**< 0.001**		
- Mean ± SD		0.6572 ± 2.1029	0.0691 ± 0.0474			
- Range		0.05–19.90	0.05–0.36			
**CRP level (mg/ml) (only available in 31 patients)**	24.0 ± 23.6	25.0 ± 25.2	22.2 ± 19.3	0.799		
**Leukocyte count (Tsd/microL)**	13.5 (IQR: 7.0–20.0)	13.4 (IQR: 6.9–19.9)	14.3 (IQR: 7.8–20.8)	0.156		
**Serum Creatinine (mg/dl)**	0.59 ± 0.16	0.59 ± 0.17	0.60 ± 0.16	0.755		
**GCS**	9.8 ± 3.4	9.0 ± 3.0	12.4 ± 3.2	< 0.001		
**Intubated and mechanically ventilated**	32 (16.6%)	28 (18.9%)	4 (8.9%)	0.114		
**Aspiration occurred**	36 (18.7%)	33 (22.3%)	3 (6.7%)	0.018		
**Recent surgery**	150 (77.7%)	116 (78.4%)	34 (75.6%)	0.692		
**Reason for NICU admission**,** n (%)** *Unplanned admission*				0.167		
- Traumatic intracranial hemorrhage	74 (38.3%)	59 (39.9%)	15 (33.3%)			
- Aneurysmal subarachnoid hemorrhage	39 (20.2%)	29 (19.6%)	10 (22.2%)			
- Spontaneous intracranial hemorrhage	36 (18.7%)	28 (18.9%)	8 (17.8%)			
- Postsurgical complications	9 (4.7%)	7 (4.7%)	2 (4.4%)			
- Central nervous system infection	2 (1.0%)	1 (0.7%)	1 (2.2%)			
*Planned admission*						
- Tumor surgery		20 (10.4%)	14 (9.5%)	6 (13.3%)		
- Degenerative spine surgery			13 (6.7%)	10 (6.8%)	3 (6.7%)	

**Fig. 1:**
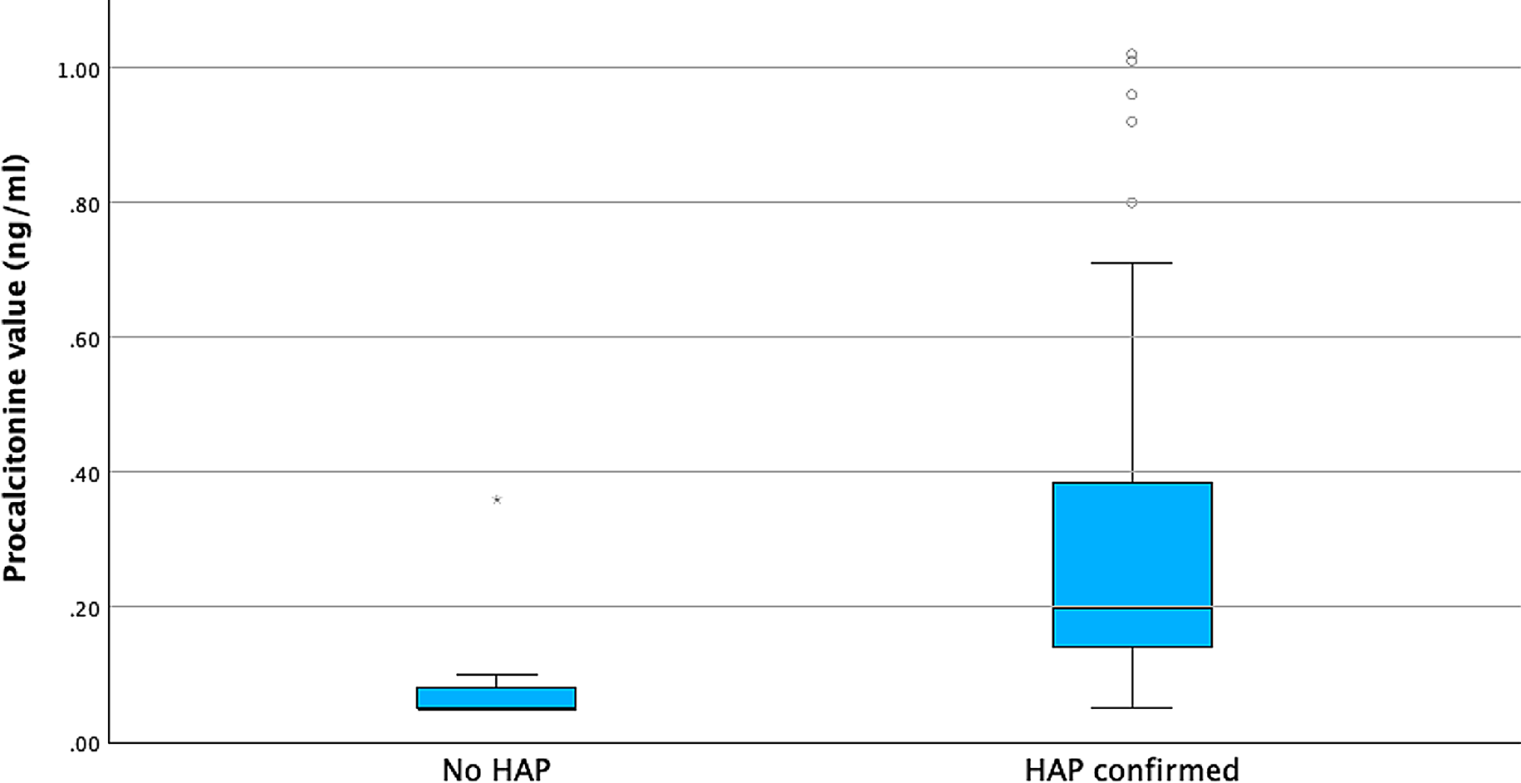
Distribution of PCT values for patients with and without confirmed HAP. Outliers were excluded from the visualization to improve clarity and focus on the central distribution of PCT levels in the two groups. However, these outliers were retained in the statistical analysis, as they represent genuine data points and may hold clinical significance

There was no significant heterogeneity observed: there was no significant association between the presence of HAP and the patient’s age (*p* = 0.419), reason for hospitalization (*p* = 0.167), or length of stay in the intensive care ward (*p* = 0.227). Furthermore, no significant differences were found in age (*p* = 0.419), length of stay (*p* = 0.227), serum creatinine (*p* = 0.755), or leukocyte count (*p* = 0.156) between HAP and non-HAP groups. Similarly, recent surgery (*p* = 0.692), reason for NICU admission (*p* = 0.167), and intubation status (*p* = 0.114) did not significantly differ between groups. In contrast, GCS scores were significantly lower in the HAP group (*p* < 0.001), and aspiration events occurred significantly more often in HAP patients (*p* = 0.018). The mean serum procalcitonin (PCT) level was significantly higher in the HAP group (0.6572 ± 2.1029 ng/mL) compared to the non-HAP group (0.0691 ± 0.0474 ng/mL) (*p* < 0.001) and ranged from 0.05 ng/ml to 19.90 ng/ml (Fig. 1).

### Diagnostic performance of biochemical markers

**Fig. 2:**
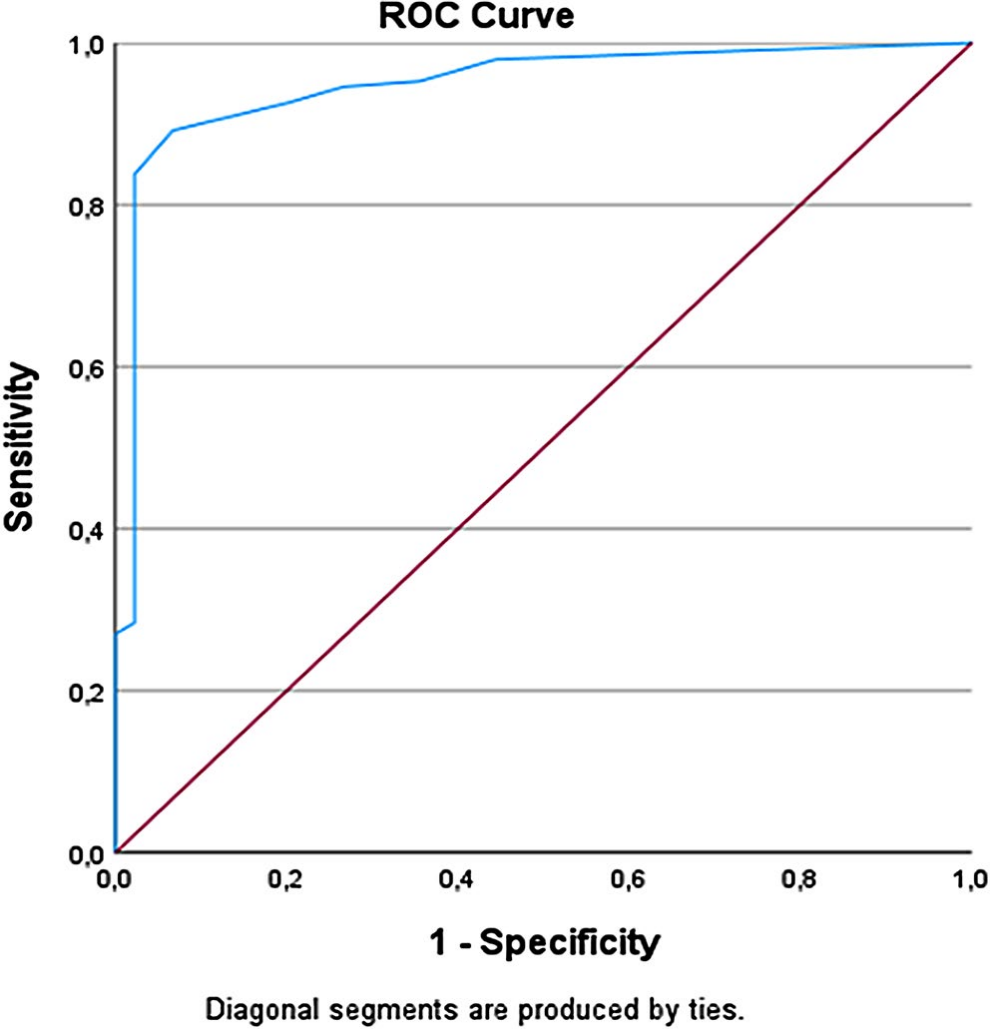
Receiver operating characteristic curve (ROC) shows an area under the curve of 0.948, corresponding with an excellent discrimination value of procalcitonin in the diagnosis of hospital-acquired pneumonia

**Fig. 3:**
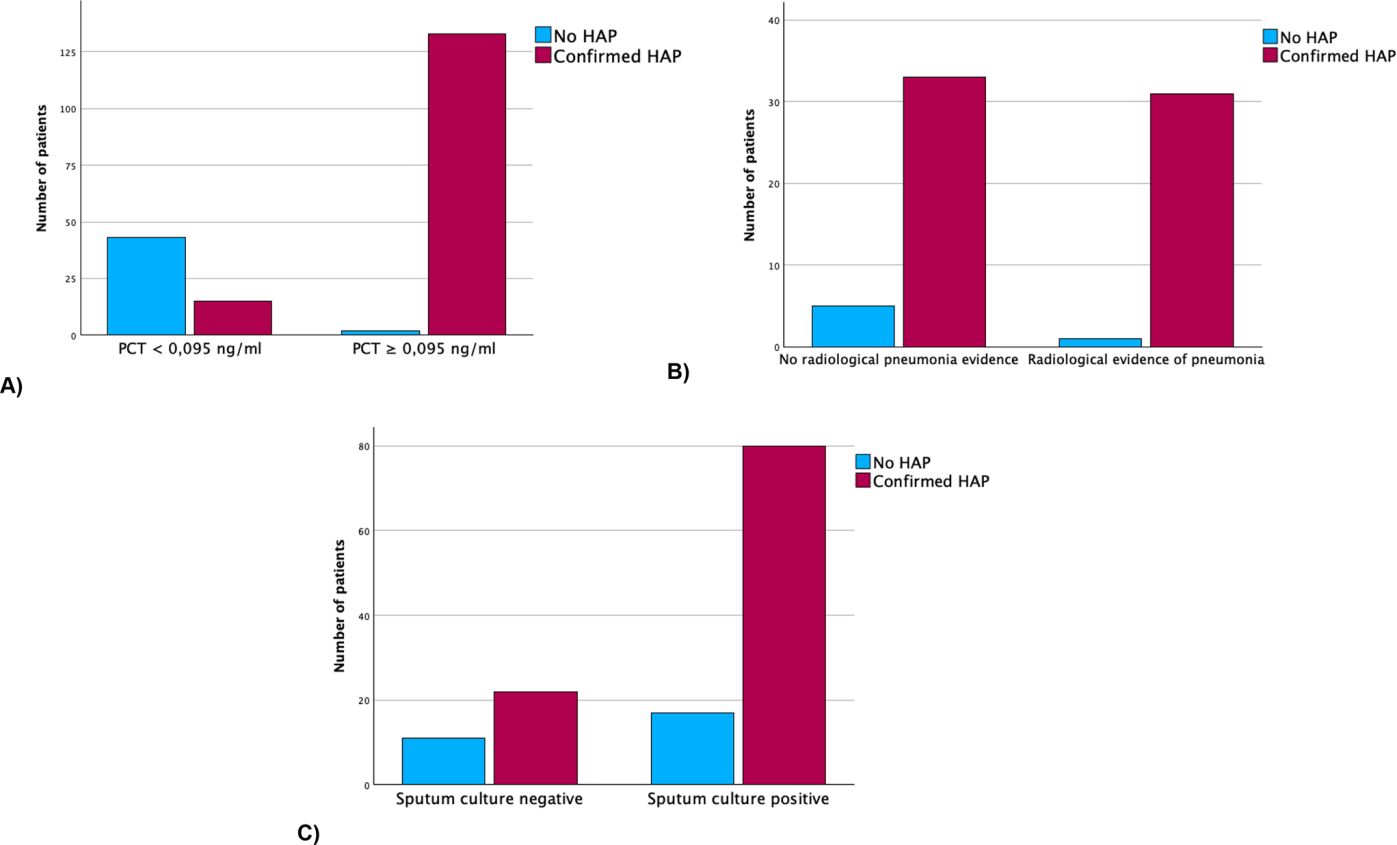
Correlation Between Hospital-Acquired Pneumonia and Serum Procalcitonin Level, radiological pneumonia evidence and sputum culture. Bars represent a 95% confidence interval. (**A**) A highly significant correlation between the presence of hospital-acquired pneumonia (HAP) and a serum procalcitonin (PCT) value of ≥ 0.095 is found. (**B**) No significant association was found between the presence of HAP and the radiological presence of pneumonia. (**C**) A relevant trend was observed; however, no significant association was found between the presence of HAP and positive sputum

The ROC Analysis showed an area under the curve of 0.948 ± 0.019 (Fig. 2). The optimal PCT value was 0,095 ng/ml, with a sensitivity of 89.2% and a specificity of 93.3% (Table 2). The chi-square test showed a highly significant correlation between the presence of HAP and a serum PCT value of ≥ 0.095 (*p* < 0.001) (Fig. 3 A). Notably, there were only 2 false positive results (1.0%) and 15 false negative results (7.8%), resulting in a positive likelihood ratio of 13.3 and a negative likelihood ratio of 0.116. The positive predictive value reached 98.5%, and the negative predictive value was 74.1%. In comparison, elevated leukocyte count was significantly associated with HAP (*p* = 0.027), with a sensitivity of 73.0% and a specificity of 44.4%. Conversely, elevated CRP (> 5 mg/L) showed a sensitivity of 95.7% and a specificity of 25.0%, though the association did not reach statistical significance (*p* = 0.089), likely due to the limited sample size.

**Table 2:** The Youden index was used to deduce the PCT serum value with the highest sensitivity and specificity. The optimal PCT value was 0,095 Ng/ml, with a sensitivity of 89.2% and a specificity of 93.3%

PCT Value	Sensitivity	Specificity	Youden-Index
0.055	0.980	0.556	0.536
0.065	0.953	0.644	0.597
0.075	0.946	0.733	0.679
0.085	0.926	0.800	0.726
0.095	0.892	0.933	0.825
0.105	0.838	0.978	0.816
0.115	0.818	0.978	0.796
0.130	0.764	0.978	0.742
0.145	0.709	0.978	0.687
0.155	0.669	0.978	0.647

### Independent predictors of elevated procalcitonin

In univariate analysis, both aspiration events (*p* = 0.024) and clinically confirmed hospital-acquired pneumonia (HAP) (*p* < 0.001) were significantly associated with elevated PCT levels (Table 3). No significant associations were observed for age, leukocytosis, GCS < 8, recent surgery, or mechanical ventilation.

**Table 3:** Univariate and multivariate logistic regression analysis for predictors of serum PCT ≥ 0.095 Ng/mL. Only clinically confirmed hospital-acquired pneumonia (HAP) remained an independent predictor in the multivariate model. Aspiration was significant in univariate analysis but lost significance after adjustment, likely reflecting its role as a risk factor for HAP

	Univariate analysis		Multivariate analysis	
Variable	P-value	OR (95% CI)	P-value	OR (95% CI)
Age (years)	0.321	1.01 (0.99–1.03)	0.537	1.01 (0.98–1.05)
Length of stay (days)	0.378	0.99 (0.97–1.01)	0.005	0.95 (0.91–0.98)
Reason for hospitalization	0.285	1.11 (0.92–1.34)	0.470	0.90 (0.68–1.20)
Recent surgery	0.728	0.88 (0.41–1.86)	0.217	0.36 (0.07–1.82)
Leukocytosis (9.8 × 10³/µL for males and 10.4 × 10³/µL for females)	0.094	1.74 (0.91–3.33)	0.926	1.06 (0.29–3.86)
Serum Creatinine	0.814	0.80 (0.12–5.18)	0.841	1.58 (0.02-141.35)
Aspiration occured	0.024	3.16 (1.16–8.60)	0.279	2.75 (0.44–17.07)
Intubated or mechanically ventilated	0.496	1.35 (0.57–3.22)	0.955	1.08 (0.09–13.35)
GCS < 8	0.529	1.34 (0.54–3.36)	0.485	0.43 (0.04–4.55)
HAP diagnosed	< 0.001	190.83 (0.58–1.20)	< 0.001	344.6 (61.95-1916.33)

However, in the multivariate model, only clinically confirmed HAP remained an independent predictor of elevated PCT (OR 344.6, 95% CI 61.95–1916.33; *p* < 0.001). Length of stay was inversely associated with elevated PCT (OR 0.95, 95% CI 0.91–0.98; *p* = 0.005), however clinically not significant. Aspiration was no longer a significant predictor in the adjusted model, suggesting that its association with elevated PCT in univariate analysis may be mediated by its role as a risk factor for developing HAP, rather than having a direct effect on PCT levels. Other variables, including leukocytosis, GCS < 8, and intubation, did not independently contribute to PCT elevation after controlling for confounders.

### Imaging and sputum culture

Chest imaging (computed tomography or RX) was performed in 70 of 193 patients (Fig. 3 B). In 31 of 64 patients with confirmed HAP, radiology showed infiltrates suggestive of pneumonia with a sensitivity of 48.4%. In contrast, in 5 of 6 patients without confirmed HAP, radiology showed no signs of pneumonia with a specificity of 83.3%. The chi-square test showed no significant association between radiological evidence of pneumonia and confirmed HAP (*p* = 0.135).

Microbiological sputum testing was performed in 130 patients (Fig. 3 C). The most frequently isolated pathogen was Staphylococcus aureus, identified in 22 of 130 patients (16.9%). Escherichia coli was found in 15 patients (11.5%), followed by Klebsiella pneumoniae in 13 patients (10.0%), and Klebsiella oxytoca in 10 patients (7.7%). The chi-square test showed no significant association between a positive sputum culture and confirmed HAP (*p* = 0.056). Among the 102 patients with confirmed HAP who underwent sputum testing, 80 (78.4%) had a positive result. Of the 28 non-HAP patients tested, 11 (39.3%) had a negative sputum culture or microscopy, yielding a specificity of 39.3%.

## Discussion

In this retrospective cohort study of NICU patients, we found that serum procalcitonin (PCT) levels with a cutoff value of 0.095 ng/mL had high sensitivity (89.2%) and specificity (93.3%) for diagnosing hospital-acquired pneumonia (HAP). This patient population’s cutoff value outperformed traditional diagnostic methods such as chest radiography and sputum cultures. The diagnostic value of procalcitonin in the diagnosis of HAP is still controversial in the literature. Our findings are consistent with previous studies that have demonstrated the utility of PCT in diagnosing lower respiratory tract infections, resulting in improved survival and lower antibiotic treatment duration [13–15]. In contrast, some studies showed a lower accuracy of procalcitonin in diagnosing pneumonia, compared to CRP and interleukin 6, and the added benefit of PCT in the NICU was viewed as minimal [9, 16, 17]. Elevated leukocyte count was significantly associated with HAP and showed a sensitivity of 73.0% and a specificity of 44.4%. In contrast, elevated CRP levels (> 5 mg/L) demonstrated a high sensitivity of 95.7% but a low specificity of only 25.0%, with data available in 31 patients. These findings suggest that while leukocytosis and CRP may support the diagnosis, they lack the diagnostic accuracy of PCT in this population. Their lower specificity limits their utility in differentiating infectious from non-infectious inflammatory states, particularly in neurosurgical ICU patients where systemic inflammation is common.

Interestingly, neither chest imaging nor positive sputum cultures were significantly associated with confirmed HAP in our cohort. This may be due to the low sensitivity of chest radiographs in critically ill patients and the challenges in obtaining high-quality sputum samples from NICU patients. These findings underscore the limitations of traditional diagnostic methods and highlight the potential of PCT as a more reliable biomarker in this context. Although PCT testing is generally more expensive than CRP, previous studies have demonstrated that its higher diagnostic accuracy may offset upfront costs by reducing unnecessary antibiotic use, hospital stays, and associated complications [13].

To our knowledge, there have yet to be studies about the optimal cut-off value of procalcitonin in diagnosing HAP. PCT is a handy tool in the neurosurgical cohort since after a standard major neurosurgical operation (e.g. tumor resection, aneurysm clipping) or aneurysmal subarachnoid hemorrhage, leukocyte and neutrophil count, as well as C-reactive Protein (CRP), are significantly elevated. In contrast, PCT levels are not elevated considerably during an ordinary postoperative course [6, 18]. Furthermore, elevated serum PCT concentrations are detectable within 6 h after an inflammatory stimulus and reach a half-maximal concentration within 8 h [2]. In contrast, CRP values reached a half-maximal concentration within 20 h. This makes PCT a tool to diagnose pneumonia faster and subsequently enables timely antibiotic therapy.

We hypothesize that the discrepancy between our results and those of select studies is due to several factors [9, 16, 17]. Firstly, there is no gold standard for diagnosing HAP, and the confirmation of HAP is often described as the presence of both infiltrates on radiological imaging in combination with clinical evidence of infection (including purulent sputum, fever and decline in oxygenation) [10]. In our series, similarly to previous literature, chest imaging sensitivity was only 48.4%^19,20^. This could be explained by a delay in developing radiological signs of pneumonia, which could be as long as 72 h, particularly on X-ray imaging [19, 20]. Moreover, it is indicated in patients with a negative initial chest imaging but a high clinical and biochemical suspicion of pneumonia to initiate an antibiotic therapy and repeat chest imaging after 48–72 h [10, 12]. Not using initial negative chest imaging as a definitive exclusion of HAP could reduce the amount of false-positive PCT tests in HAP patients with initial negative chest imaging.

Secondly, unlike previous studies that utilized procalcitonin to diagnose various bacterial infections, including urinary infections and peritonitis, our study focused solely on its application in diagnosing HAP in the NICU [13, 21]. Our study deliberately narrowed its scope to assess the diagnostic value of procalcitonin, specifically in the context of HAP in the NICU. This targeted approach allows for increased accuracy in understanding the utility of PCT in HAP diagnosis within the unique NICU setting. We acknowledge that PCT is a non-specific biomarker and can also rise in conditions such as traumatic brain injury (TBI), postsurgical inflammation, and tissue damage [6]. To assess whether PCT elevation was primarily driven by HAP, we conducted a multivariate analysis including variables such as leukocytosis, GCS < 8, intubation, recent surgery, aspiration, and creatinine. Only clinically confirmed HAP remained an independent predictor, suggesting infection was the main contributor to PCT elevation in this cohort.

Lastly, some studies have used a higher cutoff value of serum PCT to consider the result indicative of pneumonia, ranging from 0,2 ng/ml to 0,5 ng/ml^9,10,16^. It is important to note that these practices stem from expert guidelines, and comprehensive studies establishing an optimal cutoff value are currently lacking [10]. Using a higher PCT cut-off value causes a relevant decrease in the sensitivity of serum PCT value since our analysis shows that PCT values of 0,205 and 0,500 ng/ml have a sensitivity of 49,3% and 18,9%, respectively. We suggest considering a value of ≥ 0.095 ng/ml indicative of pneumonia in the diagnostic process.

We acknowledge that diagnosing pneumonia is a nuanced process, reliant on a combination of clinical, radiological and biochemical factors. It is crucial to recognize that an elevated PCT value alone is not conclusive for securing the diagnosis of HAP. This nuanced perspective underscores the complexity of HAP diagnosis. We suggest that chest infiltrations on initial imaging are not mandatory to establish a definitive HAP diagnosis. Instead, we suggest combining clinical, biochemical, and radiological signs of HAP. Furthermore, based on our findings, we suggest that PCT should not replace but rather complement traditional diagnostic tools such as chest imaging and sputum cultures. While radiography and cultures remain essential for identifying causative organisms and confirming infiltrates, their diagnostic utility is often delayed or limited in the NICU setting. The rapid availability and strong predictive value of PCT, particularly with the low cutoff value of 0.095 ng/mL, may allow for earlier clinical suspicion and targeted management in patients at risk for HAP. Therefore, we propose that PCT be considered as an adjunctive marker within existing diagnostic algorithms to support timely clinical decision-making.

To our knowledge, this is the first study to determine an optimal PCT cutoff value for diagnosing HAP, specifically in NICU patients. By focusing on this unique patient population, our study fills a critical gap in the literature and provides a foundation for improving diagnostic protocols in neurosurgical intensive care settings. Identifying an optimal PCT cutoff value at 0.095 ng/mL provides clinicians with a reliable biomarker to support the diagnosis of HAP. Implementing PCT measurement could facilitate earlier initiation of appropriate antibiotic therapy, potentially reducing morbidity, mortality, and length of NICU stay.

## Conclusion

This study demonstrates that serum PCT levels are a valuable diagnostic tool for HAP in the NICU when using a cutoff value of 0.095 ng/mL. Incorporating PCT measurements into clinical practice may enhance early detection and treatment of HAP, ultimately improving patient outcomes. These findings warrant further investigation through prospective studies to confirm their applicability and effectiveness in diverse clinical settings.

### Limitations

This study has several limitations. The retrospective design may introduce selection bias, and being a single-center study, the findings may not be generalizable to other NICUs with different patient populations or clinical practices. Additionally, the lack of a definitive gold standard for HAP diagnosis could impact the evaluation of PCT’s diagnostic performance and may introduce variability. While we included variables such as aspiration, GCS, intubation, and leukocyte count in our analysis to refine cohort characterization, we were unable to consistently distinguish ventilator-associated pneumonia (VAP) due to limitations in retrospective documentation. This may affect the level of detail achievable in pneumonia subtyping within our cohort.

## Data Availability

No datasets were generated or analysed during the current study.

## Declarations

### Reporting checklist

STARD

## Abbreviations

PCT: Procalcitonin
HAP: Hospital-acquired pneumonia
CRP: C-Reactive Protein
GCS: Glasgow coma scale
NICU: Neurosurgical Intensive Care Unit
AUC: Area under the curve
ROC: Receiver operating characteristic curve
ANOVA: Analysis of Variance Test
PPV: Positive predictive value
NPV: Negative predictive value
IQR: Interquartile range
